# Mechanism of Action and Beneficial Effects of Probiotics in Amateur and Professional Athletes

**DOI:** 10.1002/fsn3.4658

**Published:** 2024-12-12

**Authors:** Yousef Nami, Anahita Barghi, Mehdi Shahgolzari, Melika Salehian, Babak Haghshenas

**Affiliations:** ^1^ Department of Food Biotechnology, Branch for Northwest and West Region Agricultural Biotechnology Research Institute of Iran, Agricultural Research, Education and Extension Organization (AREEO) Tabriz Iran; ^2^ Institute of Agricultural Life Science Dong‐A University Busan South Korea; ^3^ Department of Medical Nanotechnology, Faculty of Advanced Medical Sciences Tabriz University of Medical Sciences Tabriz Iran; ^4^ Biotechnology Research Center Tabriz University of Medical Sciences Tabriz Iran; ^5^ Student Research Committee Kermanshah University of Medical Sciences Kermanshah Iran; ^6^ Regenerative Medicine Research Center (RMRC), Health Technology Institute Kermanshah University of Medical Sciences Kermanshah Iran

**Keywords:** athlete, beneficial effects, gut microbiome, mechanism of action, probiotics, sport

## Abstract

Probiotics are live microorganisms that, when administered in adequate amounts, provide health benefits to the host. According to the International Society of Sports Nutrition (ISSN), probiotic supplementation can optimize the health, performance, and recovery of athletes at all stages of their careers. Recent research suggests that probiotics can improve immune system functions, reduce gastrointestinal distress, and increase gut permeability in athletes. Additionally, probiotics may provide athletes with secondary health benefits that could positively affect athletic performance through enhanced recovery from fatigue, improved immune function, and maintenance of healthy gastrointestinal tract function. The integration of some probiotic strains into athletes' diets and the consumption of multi‐strain compounds may lead to an improvement in performance and can positively affect performance‐related aspects such as fatigue, muscle pain, body composition, and cardiorespiratory fitness. In summary, probiotics can be beneficial for athletes at all stages of their careers, from amateur to professional. This paper reviews the progress of research on the role of probiotic supplementation in improving energy metabolism and immune system functions, reducing gastrointestinal distress, and enhancing recovery from fatigue in athletes at different levels.

## Graphical abstract

Probiotic supplements during exercise improve athlete performance. Probiotics boost immunity, ease GI issues, and enhance gut permeability in athletes. This approach (probiotic supplementation) aids gut health and boosts immune function.

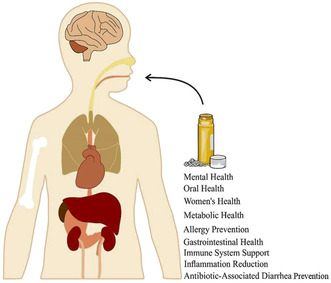

## 1 Introduction

The supplement needs depend on their energy expenditure which is influenced by exercise intensity, duration, and frequency. Competitive athletes, whether amateur or professional, may require more nutritional and sports supplements than inactive individuals (Muñoz et al. 2020). A sound nutritional plan is crucial for optimal athletic performance and requires attention before, during, and after training or competitions. Athletes can choose from a variety of foods and supplements, including probiotics, carbohydrates, and proteins, to enhance performance (Peeling et al. 2019). Dietary intake for amateur athletes, as exemplified by the Australian Guide to Healthy Eating (AGHE), is generally sufficient. However, professional athletes facing increased exercise demands or dietary restrictions should employ specific nutrition strategies to support training adaptations, optimize competition performance, and maintain overall health and well‐being (Fritzen, Lundsgaard, and Kiens 2019).

Probiotics are beneficial living microorganisms like bacteria or yeasts which, when consumed in adequate amounts, may offer health benefits. Probiotics are available in certain foods or supplements and help maintain a healthy balance of good and bad bacteria in the intestines (Iqbal et al. 2014). For example, probiotics can improve digestive health by aiding food breakdown, enhancing nutrient absorption, and preventing overgrowth of harmful bacteria (Wang and Ji 2019). They also support the immune system, as many immune cells reside in the gut. Recent studies suggest probiotics' effectiveness in managing conditions like diarrhea, IBS, and IBD, as well as reducing the severity of respiratory and urinary tract infections and vaginal yeast infections (Cruz, Ricci, and Vieira 2021). Some research even explores probiotics' potential role in mental health through the gut–brain axis. Notably, different probiotic strains have varied effects, and their suitability depends on individual health conditions. Caution is recommended for those with weakened immune systems or serious medical issues (Snigdha et al. 2022).

Probiotics offer potential benefits for athletes, especially during intense exercise and strenuous training, which can stress the immune and digestive systems (Pyne et al. 2015). Recent studies indicate that probiotic supplementation in athletes helps reduce the risk of upper respiratory tract infections, common in intense training. Probiotics strengthen the immune system, improving overall health. They also enhance gut health, alleviating gastrointestinal symptoms like bloating, gas, and diarrhea, often experienced by athletes due to factors such as high‐intensity exercise and increased caloric intake (Sivamaruthi, Kesika, and Chaiyasut 2019).

Research suggests that probiotics may enhance exercise performance (Marttinen et al. 2020). Athletes who consumed probiotics for 4 weeks showed improved time to exhaustion during high‐intensity cycling exercises. However, more research is needed to fully understand the effects of probiotics on exercise performance (Przewłócka et al. 2023). It is crucial to note that the specific strains and dosages of probiotics may vary for athletes, with individual responses differing between amateur and professional levels (Smarkusz, Ostrowska, and Witczak‐Sawczuk 2017). To make informed dietary and supplement decisions, athletes are advised to follow a well‐developed probiotics plan tailored to their specific needs and goals across different sport stages (Hannon et al. 2021). This review aims to investigate the potential benefits of probiotic supplementation for athletes, focusing on its effects on various aspects of performance, recovery, and overall health. The hypotheses to be tested include whether probiotics can enhance immune system regulation, skeletal muscle regeneration, energy metabolism, and mental well‐being in athletes. Additionally, we seek to explore their underlying mechanisms of action, evaluate their efficacy in clinical populations, and establish regulatory guidelines for safe and effective use in sports nutrition. These objectives will guide the subsequent sections of this paper, providing insights into the potential role of probiotics in optimizing athletic performance and well‐being.

## 2 Supplemental Dietary Requirements During Sports Exertion

Sports and physical activity increase the body's nutritional needs for energy production, muscle repair, and overall performance (Ghazzawi et al. 2023). Recent research, as included in “ISSN exercise & sports nutrition review update: research & recommendations,” emphasizes the role of dietary supplements in enhancing athletic performance. These supplements, marketed as ergogenic aids, often include ingredients such as creatine, protein, amino acids, caffeine, and probiotics, aiming to improve strength, endurance, and exercise efficiency (Kerksick et al. 2018). Key dietary supplements for athletes include protein for muscle repair; Branched‐Chain Amino Acids (BCAAs) for muscle recovery; creatine for short‐duration, high‐intensity exercise; carbohydrates for energy replenishment; electrolytes for intense workout support; and probiotics for overall health benefits (Rawson, Miles, and Larson‐Meyer 2018). Research in the journal *Sports* underlines the importance of dietary supplements in supporting athletes' metabolic functions, contributing to recovery, muscle growth, and energy production (Ghazzawi et al. 2023).

Research on dietary supplement consumption among athletes has indicated that a considerable number of athletes utilize dietary supplements. The prevalence of supplement use varies significantly across different sports, levels of competition, and geographical regions (Aguilar‐Navarro et al. 2021; Kovács, Liska, and Veres 2023). Studies consistently reveal that a substantial percentage of athletes consume dietary supplements, with this prevalence ranging from 40% to over 90%, contingent upon the population studied (Jagim et al. 2023; Vento and Wardenaar 2020). Elite athletes often exhibit higher rates of supplement use compared to recreational athletes (McDaid et al. 2023). The effectiveness of dietary supplements in athletes is a subject of mixed research findings. While some supplements, such as creatine and caffeine, have been demonstrated to have performance‐enhancing effects, others may not offer significant benefits (Arieli and Lahav 2016; Trexler and Smith‐Ryan 2015). Furthermore, there are ongoing concerns regarding the safety and purity of supplements, as some products may contain banned substances or contaminants (Cadwallader 2022).

Numerous investigations have been conducted into the ingestion of dietary supplements by athletes, examining facets such as the prevalence, the variety of supplements employed, and the motivations driving their use (Daher, Mallick, and El Khoury 2022). Research on female elite footballers has indicated that the propensity to consume sports supplements is correlated with age, with older athletes exhibiting a higher likelihood of supplementation (Molina‐López et al. 2024). Another investigation revealed that approximately two‐thirds of elite track and field athletes competing in world championships consumed one or more dietary supplements, with usage increasing with age and being more prevalent among female athletes than their male counterparts (Tscholl et al. 2010). A survey of U.S. collegiate athletes disclosed that 41.7% utilized protein products, 28.6% consumed energy drinks and shots, 14.0% used creatine, and 12.1% opted for amino acids (Hoyte, Albert, and Heard 2013). A synthesis of studies on adolescent athletes' use of performance‐enhancing substances identified protein, creatine, and caffeine as the most frequently consumed substances (Frączek et al. 2016). The consumption of sports drinks, sports bars, and products containing omega‐3 fatty acids was also documented in various studies (Knapik et al. 2016). An investigation into professional team sport athletes demonstrated that both objective knowledge and self‐perceived competence played a role in the decision to use dietary supplements (Sekulic et al. 2019). A survey across U.S. colleges found that 66% of student athletes use dietary supplements, primarily for enhanced muscle strength (20% of users), performance enhancement (19% of users), and increased endurance (7% of users) (Lieberman et al. 2015). International surveys on elite track and field athletes and U.S. military personnel reveal widespread dietary supplement usage, with protein, energy drinks, and creatine being popular choices (LaBotz et al. 2016). Further research indicated that the frequency of dietary supplement consumption differed across various sports, with ice hockey, wrestling, and baseball among men, and volleyball, swimming, and ice hockey among women, showing the highest rates of usage (Kovács, Liska, and Veres 2023; Knapik et al. 2016). A study on young elite athletes at the national level revealed a high prevalence of supplement use but a variable level of understanding regarding their effects and the ethical considerations involved (Jovanov et al. 2019). The sources of information about supplements and the reasons for their use were also explored, with athletes frequently turning to coaches, teammates, and the internet for guidance (Lopes et al. 2024). Collectively, these studies offer a thorough examination of the patterns of consumption, the motivations, and the implications of dietary supplement use among athletes, underscoring the necessity for informed and ethical consumption practices.

While there are no specific micronutrient recommendations for athletes, a balanced diet that meets their energy needs is crucial. Personalized recommendations from sports nutritionists are advised before starting any new supplement regimen, as individual needs may vary. Supplements should complement a balanced diet and be used judiciously based on athletic goals and requirements (Hannon et al. 2021; Ghazzawi et al. 2023).

## 3 General Health Benefits of Probiotics and Their Mechanisms of Action

**FIGURE 1:**
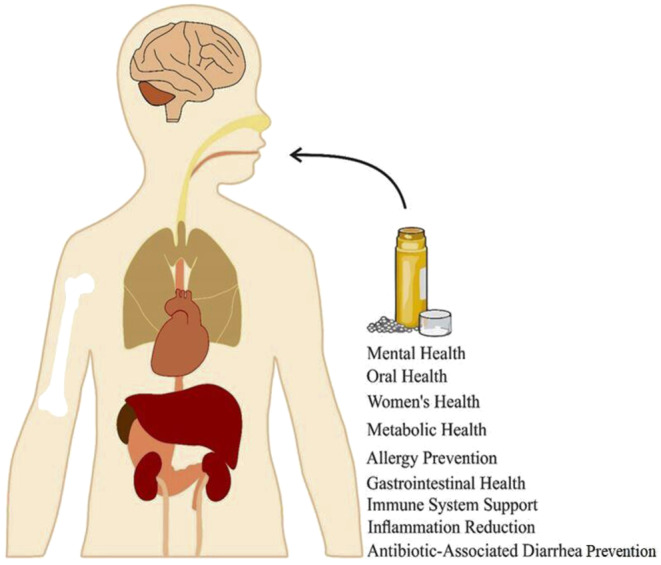
Health benefits associated with probiotics.

Probiotics offer a plethora of health benefits by positively influencing the balance and activity of the gut microbiota, a diverse community of microorganisms in the digestive tract (Dahiya and Nigam 2022). Notable among these benefits (Figure 1) is their role in maintaining the balance of the gut microbiota by promoting the growth of beneficial bacteria and inhibiting the proliferation of harmful microorganisms. Additionally, they have garnered attention for their potential in managing and preventing various digestive issues such as irritable bowel syndrome (Quin et al. 2018), inflammatory bowel diseases (IBD), and diarrhea (Ahlawat and Singh 2023). Additionally, probiotics possess the ability to stimulate the immune system, thereby triggering an increased production of antibodies and the activation of immune cells. This immune modulation can lead to enhanced defense against infections and overall improvement in immune function. Specifically, certain strains of probiotics, such as *Lactobacillus*, have shown promise in preventing and managing vaginal infections by fostering a healthy balance of microorganisms in the urogenital tract (Kim et al. 2022). They can also help prevent or reduce antibiotic‐associated diarrhea by restoring gut microbiota balance. Antibiotics, while beneficial in treating infections, can disrupt the delicate equilibrium of the gut microbiota, often resulting in antibiotic‐associated diarrhea. Probiotics have emerged as a potential solution to mitigate or prevent the severity of diarrhea in individuals undergoing antibiotic treatment (Mekonnen et al. 2020).

Furthermore, the gut microbiota communicates bidirectionally with the brain through the gut–brain axis. Probiotics have the capacity to influence this axis, potentially exerting positive effects on mood and mental well‐being (Appleton 2018). They also modulate immune responses to reduce the risk of allergic conditions like eczema, especially in high‐risk infants (Fiocchi et al. 2015), improve glucose metabolism, and contribute to favorable changes in lipid profiles, benefiting individuals with insulin resistance or type II diabetes (Kasińska and Drzewoski 2015). They may also aid in reducing inflammation in the gut and other parts of the body, assisting in the management of inflammatory conditions (Patra et al. 2022). Particularly, strains such as *Lactobacillus* and *Bifidobacterium* may maintain oral health by reducing the risk of gum disease and promoting a healthy balance of oral microorganisms (Terai et al. 2015).

It is crucial to note that the effectiveness of probiotics varies depending on the strain, each with its distinct mechanisms of action. Individual responses may vary, and further research is needed to determine the optimal strains, dosages, and duration of probiotic supplementation (Dimidi, Scott, and Whelan 2020). Numerous probiotic strains have been demonstrated to confer general health benefits (Capurso 2016). *Lactobacillus acidophilus* is recognized for its capacity to enhance gut health, bolster immune function, and mitigate symptoms of lactose intolerance (Bull et al. 2014). *Lactobacillus rhamnosus* is effective in the prevention and treatment of diarrhea, particularly antibiotic‐associated diarrhea, and has also been associated with improvements in mental health conditions such as anxiety and depression (Petrova, Reid, and Ter Haar 2021; Stage et al. 2020). *Lactobacillus casei* contributes to digestive health and has been shown to diminish the severity of diarrhea (Hill et al. 2018). *Lactobacillus plantarum* supports gut health, enhances immune function, and has been linked to the reduction of symptoms associated with inflammatory bowel disease (IBD) (Seddik et al. 2017). *Lactobacillus reuteri* is effective in alleviating symptoms of irritable bowel syndrome (Cronin et al. 2022) and improving gut health (Wang et al. 2024a). *Bifidobacterium bifidum* promotes gut health, enhances immune function, and has been associated with the reduction of IBS symptoms (Marcos‐Fernández et al. 2023). *Bifidobacterium lactis* is known for its ability to improve gut health, enhance immune function, and reduce IBS symptoms (Cheng, Laitila, and Ouwehand 2021). *Saccharomyces boulardii* is effective in preventing and treating various forms of diarrhea, including antibiotic‐associated diarrhea and traveler's diarrhea (Gopalan et al. 2023). These strains are commonly present in probiotic supplements and fermented foods, and their various health benefits have been extensively studied.

**FIGURE 2:**
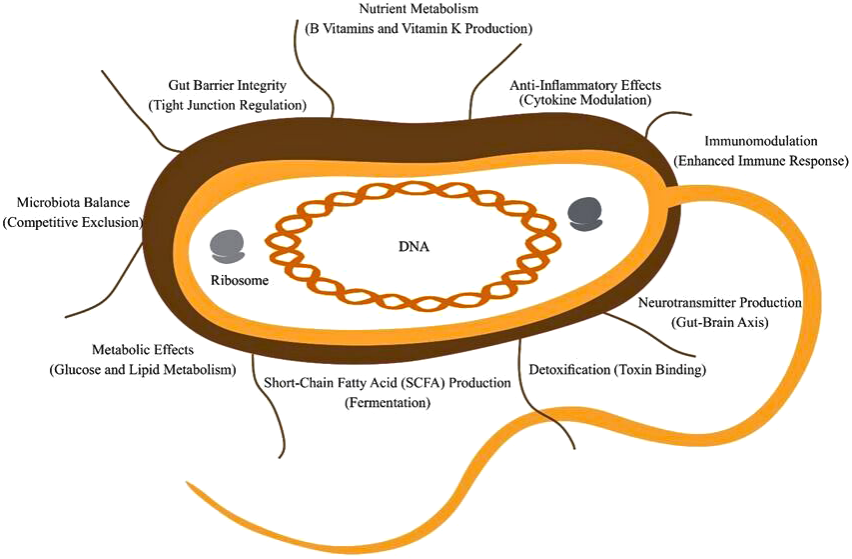
Key action mechanisms of probiotics.

Probiotics exert their diverse beneficial effects through various mechanisms, with specific strains playing a pivotal role in shaping their actions. While these mechanisms (Figure 2) predominantly unfold in the gastrointestinal tract, their influence can extend to other bodily systems (Sánchez et al. 2017). One key action mechanism involves probiotics competing with harmful microorganisms for space and nutrients, thereby preventing the overgrowth of pathogenic bacteria and maintaining microbial balance (Kober et al. 2022). Additionally, they regulate tight junctions between intestinal cells, enhancing gut barrier integrity and reducing the risk of harmful substances crossing into the bloodstream (Shehata et al. 2022). Probiotics also interact with the immune system, stimulating antibody and immune cell production to bolster the body's defense against infections (Hachimura, Totsuka, and Hosono 2018). Furthermore, they modulate cytokine production, thereby curbing inflammation in the gut and other bodily regions (Plaza Díaz et al. 2014).

Moreover, probiotic fermentation of dietary fibers yields short‐chain fatty acids (SCFAs) with anti‐inflammatory properties, which contribute to intestinal health (Bongiovanni, Yin, and Heaney 2021). Certain probiotics also synthesize B vitamins and vitamin K, thereby influencing the host's overall nutrient status (Celebi et al. 2023). Additionally, probiotics can bind to and neutralize toxins produced by harmful bacteria, thus preventing their absorption and reducing toxin‐related issues (Giridhar et al. 2024).

Some probiotics have been associated with improved glucose metabolism and lipid profiles, potentially offering benefits to individuals with metabolic disorders (Wang et al. 2021). Furthermore, probiotics influence the gut–brain axis by producing neurotransmitters like serotonin and gamma‐aminobutyric acid (GABA), which may impact mood and cognitive function (Bhatia et al. 2023).

## 4 Probiotic Consumption in Athletes

Probiotics, which are live microorganisms that offer health benefits when consumed appropriately, have garnered significant interest in the realm of sports nutrition. Athletes stand to gain from probiotics in several ways. Regular exercise can disturb the balance of the gut microbiota, leading to dysbiosis. Probiotics aid in restoring a healthy microbial equilibrium, thereby reducing the risk of gastrointestinal issues (Dahiya and Nigam 2023). Furthermore, athletes who are particularly susceptible to infections may find benefit in probiotics that stimulate the production of immune cells and enhance defense mechanisms (Pyne et al. 2015). Certain strains of probiotics, such as *Lactobacillus* and *Bifidobacterium*, possess anti‐inflammatory properties, which could potentially alleviate exercise‐induced inflammation (Sagar et al. 2014). Moreover, probiotics assist in the breakdown of complex carbohydrates and fibers, improving digestibility and absorption, consequently enhancing energy availability during exercise (Hornung et al. 2018).

Athletes can consume probiotic supplements in various sports, particularly those that require high endurance, strength, and recovery (Wiącek and Karolkiewicz 2023). Probiotics are beneficial for athletes in general by improving gut health, which can indirectly enhance overall performance. They help in maintaining a healthy gut microbiota, which is crucial for digestion, absorption of nutrients, and immune function (Marttinen et al. 2020). Probiotics have been shown to improve endurance performance by reducing fatigue, promoting post‐exercise recovery, enhancing nutrient absorption and energy supply, and improving immune function and gut health (Zhang et al. 2024). Studies have indicated that probiotics can help endurance athletes by delaying the onset of fatigue, improving aerobic and anaerobic capacity, and reducing inflammation and oxidative stress (Díaz‐Jiménez et al. 2021; Cox et al. 2010). Probiotics can also benefit athletes in strength sports by improving muscle gain, insulin sensitivity, and reducing inflammation. They may help in muscle recovery and reducing muscle damage post‐exercise (Rawson, Miles, and Larson‐Meyer 2018).

Probiotic strains are frequently utilized by athletes to augment their health and performance. The genera *Lactobacillus* and *Bifidobacterium* are widely employed in probiotic formulations for athletes due to their extensive benefits in gastrointestinal health, immune function, and overall well‐being (Sivamaruthi, Kesika, and Chaiyasut 2019; Miles 2020). Notably, *Lactobacillus helveticus* Lafti L10 has demonstrated significant enhancement of immune status in elite athletes when administered at a dosage of 2 × 10^10^ CFU daily for a period of 14 weeks (Michalickova et al. 2016). Additionally, *Lactobacillus fermentum* E3 and E18 are known to produce antioxidants such as glutathione, which may help mitigate oxidative stress in athletes (Kullisaar et al. 2002). *L. plantarum* PS128 has been shown to improve both anaerobic and aerobic endurance, reduce fatigue, and alleviate inflammation and oxidative stress (Fu et al. 2021; Huang et al. 2019). *Clostridium butyricum* is recognized for its production of short‐chain fatty acids, which can benefit gut health and overall athletic performance (Cassir, Benamar, and La Scola 2016; Cazzaniga et al. 2024). *Bifidobacterium longum* OLP‐01, isolated from an Olympic weightlifting champion, has been formulated to enhance athletic performance and is now available in probiotic supplements (Lin et al. 2022). *Veillonella atypica* , identified in the gut microbiota of elite athletes, is believed to contribute to improved physical performance and is currently being developed into probiotic supplements (Scheiman et al. 2019). These strains are typically consumed in the form of supplements, powders, or are incorporated into foods such as yogurt and fermented products to support the health and performance of athletes. For professional athletes, recommendations include *L. rhamnosus* (LGG) and *Bifidobacterium animalis* ssp. *Lactis* (BB‐12) strains (Poutsiaka et al. 2017). Proper labeling, which specifies the genus, species, strain name, and the expected quantity at the end of the supplement's shelf life, is crucial. It is important to note that the effects of probiotics can vary depending on the strains and doses used, underscoring the significance of personalized choices and seeking professional consultation (Jäger et al. 2019a).

## 5 Health Benefits of Probiotics in Athletes

### 5.1 Gut Health

**Table:** 

Key point 1: Benefits of probiotics on athlete gut health
Healing GI distress Increased nutrient absorption Hydration and electrolyte balance Improved nutrient utilization Reduced inflammation

The link between gut health and probiotics (Table 1) is of significant interest among athletes due to its profound influence on performance, recovery, and overall well‐being (Marttinen et al. 2020). Probiotics play a crucial role in maintaining a balanced gut microbiome, which is essential for digestive health. Among the benefits they offer athletes is alleviating common gastrointestinal issues experienced during intense exercise, such as bloating and cramping (Pyne et al. 2015). Additionally, a healthy gut microbiome influenced by probiotics facilitates optimal nutrient absorption, a key factor in effective recovery (Wang et al. 2022).

**TABLE 1:** Key ways in which probiotics play a role in athletes' health.

Health benefit	Key ways in which probiotics play a role in health
Gut health	Healing GI distress, increased nutrient absorption, hydration and electrolyte balance, improved nutrient utilization, reduced inflammation
Intestinal permeability	Tight junction regulation, mucin production, inflammation reduction, short‐chain fatty acid (SCFA) production, immunomodulation, prevention of pathogen adhesion
Immune function	Enhanced antigen presentation, activation of immune cells, cytokine production, immunoglobulin production, reduced inflammation, protection against upper respiratory tract infections (URTIs), stress response and gut‐associated lymphoid tissue (GALT) modulation, recovery from exercise‐induced immune suppression
Inflammation and oxidative stress	Modulation of immune response, enhancement of gut barrier function, reduced intestinal permeability, production of short‐chain fatty acids (SCFAs), inhibition of NF‐κB activation, antioxidant properties, systemic effects beyond the gut
Psychological stress	Gut–brain axis communication, neurotransmitter production, inflammation reduction, hormonal regulation, vagus nerve stimulation, immune system modulation
Antioxidant activity	Production of antioxidant molecules, scavenging free radicals, enhancement of host antioxidant defenses, reduced oxidative stress, protection against lipid peroxidation, fermentation, and short‐chain fatty acids (SCFAs)
Nutrient absorption	Gut microbiota balance, improved gut barrier function, enhanced digestive enzyme activity, short‐chain fatty acid production, optimized nutrient metabolism
Gut–brain axis	Neurotransmitter production, immunomodulation, short‐chain fatty acid production, hormonal regulation, reduced inflammation
Bone health	Nutrient absorption, vitamin production, reduced inflammation, short‐chain fatty acids (SCFAs), hormonal regulation
Recovery and muscle soreness	Reduced inflammation, enhanced immune function, gut–brain axis interaction, improved nutrient absorption, regulation of gut permeability
Skeletal muscle metabolism	Inflammation modulation, short‐chain fatty acids (SCFAs), insulin sensitivity, muscle protein synthesis, hormonal regulation
Fat metabolism	Microbiota composition, short‐chain fatty acids (SCFAs), inflammation reduction, insulin sensitivity, hormonal regulation, energy expenditure
Body weight	Regulation of appetite, modulation of metabolism, influence on fat mass, anti‐inflammatory effects, short‐chain fatty acid production, improved nutrient absorption

Furthermore, probiotics may help create a balanced gut environment, potentially enhancing fluid and electrolyte absorption, which is vital for athletes (Ribeiro et al. 2021). Moreover, by improving nutrient absorption, including vitamins and minerals, probiotics support athletes' nutritional requirements (Judkins et al. 2020). They may also play a role in modulating gut inflammation, potentially alleviating exercise‐induced inflammation and related symptoms (Park et al. 2018).

In conjunction with probiotics, the role of diet in post‐exercise recovery is paramount for athletes. Proper nutrition plays a crucial role in replenishing energy stores, repairing muscle damage, and supporting immune function after strenuous activity. Carbohydrates are essential for replenishing glycogen stores, while protein aids in muscle repair and growth. Healthy fats help reduce inflammation and support overall cellular function. Additionally, adequate hydration is vital for optimizing recovery processes (Thomas, Erdman, and Burke 2016).

Athletes should focus on consuming a balanced diet consisting of whole foods, including lean proteins, complex carbohydrates, healthy fats, fruits, and vegetables. Timing meals and snacks around exercise sessions is also important to maximize nutrient delivery and recovery. Incorporating nutrient‐dense foods into post‐exercise meals and snacks can promote faster recovery and enhance overall performance. Considering both probiotics and dietary strategies in post‐exercise recovery can provide athletes with comprehensive support for optimizing performance and well‐being. Therefore, it is crucial for athletes and sports professionals to emphasize the role of diet alongside other interventions for effective recovery and long‐term athletic success (Potgieter 2013).

The impact of the gut microbiome on various aspects of athletic health, including performance, is widely acknowledged. Probiotic supplementation positively affects meeting the demands of exercise training and competition. Athletes often encounter the challenge of maintaining optimal gut function during exercise, when blood flow is redirected, and sympathetic activation is heightened (Marttinen et al. 2020). It is important to note that individual responses to probiotics can vary depending on factors such as baseline gut health, specific strains, and variations in individual gut microbiota. Therefore, ongoing research in the athletic context focuses on areas such as selecting probiotic strains, timing supplementation in relation to exercise, and determining appropriate dosages (Pyne et al. 2015).

The gut microbiome, a diverse ecosystem of microorganisms in the gastrointestinal tract, plays a crucial role in overall health, influencing digestion, immune system, mental health, and metabolism (Milroy et al. 2014). Maintaining a healthy gut microbiome involves having a diverse range of beneficial bacteria, achievable through a diet rich in fiber, whole grains, fruits, vegetables, and fermented foods (Dahiya and Nigam 2022). Research suggests that athletes generally exhibit a more diverse gut microbiome compared to non‐athletes, with certain bacterial strains associated with health found in greater abundance (Quin et al. 2018). Athletes' higher dietary fiber intake contributes to this diversity, supporting better digestion, nutrient absorption, and immune function. Studies indicate that the gut microbiome can impact athletes' exercise capacity, inflammation, and energy utilization during physical activity, potentially enhancing endurance and recovery (Ahlawat and Singh 2023). Exercise itself influences the gut microbiome, with prolonged sessions leading to changes in bacterial composition. Strategies to optimize the gut microbiome for athletic performance include maintaining a balanced diet, incorporating fiber‐rich foods, and considering probiotics or fermented foods. However, the impact of the gut microbiome on athletic performance is complex and individualized, influenced by factors such as genetics, diet, and training regimen. Ongoing research aims to uncover specific mechanisms and strategies to optimize the gut microbiome for athletic success (Mekonnen et al. 2020).

Recent studies have identified various probiotic strains as advantageous for maintaining gut health in athletes (Pyne et al. 2015). *L. plantarum* has demonstrated efficacy in enhancing both anaerobic and aerobic endurance, mitigating fatigue, and reducing inflammation and oxidative stress leading to gut health in athletes (Huang et al. 2020b). *L. acidophilus* supports gut health and immune function, consequently decreasing the prevalence of gastrointestinal symptoms and upper respiratory tract infections (Li et al. 2022). *L. casei* has been linked to improved immune function and a lowered incidence of gut tract infections (Jespersen et al. 2015). *B. bifidum* is recognized for its role in promoting gut health and strengthening immune responses, which is particularly beneficial for athletes engaged in intensive training regimes (Arboleya et al. 2016). *Streptococcus thermophilus* , often utilized in conjunction with other probiotics, aids in supporting gut health and immune function, thereby enhancing overall well‐being in athletes (Pane et al. 2018).

### 5.2 Intestinal Permeability

**Table:** 

Key point 2: Key ways in which probiotics may contribute to a decrease in intestinal permeability during sport
Tight junction regulation Mucin production Inflammation reduction Short‐chain fatty acid (SCFA) production Immunomodulation Prevention of pathogen adhesion

Intestinal permeability, or “leaky gut,” can result in health issues, especially in athletes exposed to intense exercise and stress (Ribeiro et al. 2021). Probiotics play a role in maintaining the integrity of the intestinal barrier, potentially reducing leakage and inflammation. Strenuous physical activity can increase gut permeability, leading to gastrointestinal (GI) distress, but probiotics are studied for their potential to modulate intestinal permeability. Their relevant mechanisms of action are summarized below.

Tight junction regulation: Tight junctions, which are protein complexes connecting intestinal epithelial cells, serve as a barrier controlling the passage of molecules. Probiotics have shown potential in regulating the integrity of these tight junctions, thereby enhancing barrier function and reducing permeability.

- Mucin production: Mucin, glycoproteins that create a protective layer covering the intestinal epithelium, plays a crucial role in gastrointestinal health. Certain strains of probiotics, notably Lactobacillus and Bifidobacterium, have been demonstrated to stimulate mucin production. This stimulation contributes significantly to the maintenance of the mucus layer and the overall enhancement of barrier function in the intestine.
- Inflammation reduction: Chronic inflammation is known to exacerbate intestinal permeability, leading to various gastrointestinal issues. Probiotics are recognized for their anti‐inflammatory properties, which aid in mitigating inflammation within the gut. By doing so, probiotics play a vital role in supporting the preservation of a healthy intestinal barrier, thereby reducing the risk of increased permeability and associated complications.
- Short‐chain fatty acid (SCFA) production: Probiotic bacteria have the ability to ferment dietary fiber, resulting in the production of SCFAs as metabolic byproducts. Among these SCFAs, butyrate stands out for its significant role in supporting the integrity of the intestinal barrier and enhancing the stability of tight junctions.
- Immunomodulation: Probiotics engage with immune cells within gut‐associated lymphoid tissue, influencing the immune response. This modulation of immune activity by probiotics has the potential to prevent excessive immune activation, which might otherwise compromise the integrity of the intestinal barrier.
- Prevention of pathogen adhesion: Probiotics have the capability to compete with pathogenic bacteria for binding sites within the intestines (Ribeiro et al. 2021). This competitive interaction impedes the adhesion of harmful microorganisms to the intestinal epithelium. As a result, probiotics play a crucial role in reducing gut permeability and maintaining intestinal health (Wu et al. 2022a).

Studies, particularly on *Lactobacillus* and *Bifidobacterium* strains, suggest that probiotic supplementation can decrease the frequency and severity of intestinal permeability during training, with multi‐strain probiotics showing higher effectiveness (Miles 2020). Several probiotic strains, including *L. plantarum* , *L. fermentum* , *L. acidophilus* , *L. casei* , and *B. bifidum* , have been demonstrated to reduce intestinal permeability, commonly referred to as “leaky gut,” in athletes. These strains contribute to maintaining the integrity of the gut lining, which is essential for overall health and performance in athletes (Smarkusz, Ostrowska, and Witczak‐Sawczuk 2017; Bubnov et al. 2018; Jäger et al. 2019b).

The duration of supplementation is a crucial factor. While evidence supports probiotics' potential benefits, effects depend on strains, dosage, and individual factors. Probiotics should be part of a holistic approach to gut health in athletes, involving a balanced diet, sufficient fiber, and a healthy lifestyle (Marttinen et al. 2020).

### 5.3 Immune Function

**Table:** 

Key point 3: Key ways in which probiotics may play a role in immune system modulation in athletes
Enhanced antigen presentation Activation of immune cells Cytokine production Immunoglobulin production Reduced inflammation Protection against upper respiratory tract infections (URTIs) Stress response Gut‐associated lymphoid tissue (GALT) Recovery from exercise‐induced immune suppression

Intense physical activity can temporarily suppress the immune system in athletes, making them more susceptible to infections (Simpson et al. 2020). Probiotics are being studied for their potential to modulate the immune system in athletes. Possible mechanisms include enhanced antigen presentation, activation of immune cells, cytokine production, immunoglobulin production, reduced inflammation, protection against upper respiratory tract infections (URTIs), stress response mitigation, and recovery from exercise‐induced immune suppression (Tavares‐Silva et al. 2021).

Probiotics have the capacity to stimulate the activity of immune cells, including dendritic cells, which play a crucial role in presenting antigens to other immune cells. This heightened antigen presentation is believed to enhance the immune response. Additionally, probiotics can activate various immune cells such as macrophages, natural killer (NK) cells, and T lymphocytes, leading to an increased ability of the immune system to recognize and combat potential pathogens. Furthermore, probiotics have been shown to influence the production of cytokines, pivotal signaling molecules that regulate the immune response. The modulation of cytokine production by probiotics can help maintain a balanced immune response. Moreover, probiotics may stimulate the production of immunoglobulins, particularly secretory IgA, which is essential for mucosal immunity. This is significant for preventing infections at mucosal surfaces, including the respiratory and gastrointestinal tracts. Chronic inflammation can detrimentally affect immune function. Probiotics, with their anti‐inflammatory properties, have the potential to reduce overall inflammation, thereby supporting a more effective immune response.

Athletes, particularly those engaged in endurance sports, often face susceptibility to upper respiratory tract infections (URTIs). Probiotics may play a role in decreasing both the incidence and severity of URTIs by bolstering immune defenses in the respiratory tract (Heimer et al. 2022). Intense and prolonged physical exercise can induce stress on the body, impacting immune function. Probiotics may aid in mitigating the stress response and maintaining a balanced immune system in athletes. Importantly, probiotics exert their effects not only in the gut but also in gut‐associated lymphoid tissue (GALT), a significant component of the immune system. Modulating the GALT can have systemic effects on immune function. Probiotics may thus contribute to the restoration of immune function after periods of intense exercise, assisting athletes in recovering from exercise‐induced immune suppression more efficiently (Łagowska and Bajerska 2021).

Numerous probiotic strains have been recognized for their ability to enhance immune function and exert immune‐modulating effects in athletes, which is crucial for maintaining optimal athletic performance (Jäger et al. 2019b). *L. plantarum* strains, such as *L. plantarum* PS128 and *L. plantarum* Tana, have been shown to enhance exercise performance, reduce fatigue, and alleviate inflammation and oxidative stress in triathletes (Huang et al. 2019). Conversely, *L. fermentum* VRI‐003 has been investigated for its effects on mucosal immunity in endurance athletes, indicating potential benefits for immune function in this population (Cox et al. 2010). Additionally, *L. acidophilus* , often paired with *B. animalis* subsp. *lactis*, has been demonstrated to support immune function in athletes (Smarkusz, Ostrowska, and Witczak‐Sawczuk 2017; Rubin et al. 2022). Furthermore, *L. rhamnosus* is notable for its ability to enhance immune function, which is particularly advantageous for athletes at higher risk due to intensive training and competition schedules (Sivamaruthi, Kesika, and Chaiyasut 2019). *B. animalis* subsp. *lactis*, frequently combined with other probiotics like *L. acidophilus* , has been utilized in studies to improve immune function in marathon runners (Sivamaruthi, Kesika, and Chaiyasut 2019). Moreover, *B. bifidum* has been investigated for its role in modulating the immune system, indirectly supporting athletic performance by reducing the risk of gastrointestinal issues and infections (Gavzy et al. 2023).

### 5.4 Inflammation and Oxidative Stress

**Table:** 

Key point 4: Overview of how probiotics may exhibit anti‐inflammatory and oxidative stress effects
Modulation of the immune response Enhancement of gut barrier function Reduced intestinal permeability Production of short‐chain fatty acids (SCFAs) Inhibition of NF‐κB activation Antioxidant properties Systemic effects beyond the gut

Intense physical exercise can induce inflammation and oxidative stress in athletes, affecting recovery and performance (Kruk, Aboul‐Enein, and Duchnik 2021). Probiotics are being investigated for their potential to mitigate exercise‐induced inflammation and oxidative stress. Probiotics may have anti‐inflammatory properties by modulating the immune response, enhancing gut barrier function, reducing intestinal permeability, producing short‐chain fatty acids, inhibiting NF‐κB activation, and exerting antioxidant effects (Huang et al. 2019). Certain probiotic strains, such as *Lactobacillus* and *Bifidobacterium*, have shown promise in down‐regulating pro‐inflammatory cytokines and promoting anti‐inflammatory responses (Falalyeyeva et al. 2017). *L. plantarum* is acknowledged for its capacity to improve exercise performance by reducing inflammation and oxidative stress (Huang et al. 2019). *L. fermentum* has been the subject of research into its impact on oxidative stress and its potential to alleviate inflammation (Li et al. 2021a). *L. acidophilus* , frequently administered in conjunction with *B. animalis* subsp. *lactis*, supports immune function and contributes to the mitigation of inflammation (Li et al. 2019). *B. bifidum* plays a role in modulating the immune system, indirectly supporting athletic performance by reducing inflammation and oxidative stress (Gavzy et al. 2023).

Probiotics contribute to gut health by supporting tight junction protein production and reducing intestinal permeability. The production of short‐chain fatty acids, inhibition of NF‐κB activation, and antioxidant properties further contribute to anti‐inflammatory effects. Probiotics may influence systemic inflammation beyond the gut (Obianwuna et al. 2023). Clinical studies have explored the anti‐inflammatory effects of probiotics in various conditions, suggesting potential benefits (Guo et al. 2022). In athletes, probiotic supplementation has been associated with reduced markers of inflammation. However, responses are strain‐specific, and individual variations may occur based on factors like diet and baseline gut microbiota composition. Athletes should consider probiotic strains with demonstrated anti‐inflammatory and antioxidant properties, but further research is needed to establish specific protocols for optimal use in this context (Pyne et al. 2015).

### 5.5 Psychological Stress

**Table:** 

Key point 5: Pathways through which probiotics might influence stress and mental well‐being
Gut–brain axis communication Neurotransmitter production Inflammation reduction Hormonal regulation Vagus nerve stimulation Immune system modulation

The potential link between probiotic consumption and a reduction in psychological stress in athletes is a subject of ongoing research. Probiotics may influence stress and mental well‐being through various pathways, including gut–brain axis communication, neurotransmitter production, inflammation reduction, hormonal regulation, vagus nerve stimulation, and immune system modulation (Liu, Cao, and Zhang 2015).

Probiotics can impact the gut–brain axis, influencing mood and the stress response through the production of neurotransmitters and short‐chain fatty acids (Bienenstock, Kunze, and Forsythe 2015). Some probiotics modulate neurotransmitter production, potentially regulating mood and stress. The anti‐inflammatory effects of probiotics may indirectly contribute to a balanced stress response and influence hormonal regulation, including cortisol levels. Additionally, probiotics might stimulate the vagus nerve, leading to a calming effect, and modulate the immune system, associated with improved mental well‐being (Clark and Mach 2016).

Research suggests that probiotics can help improve mental health by reducing stress and anxiety, improving cognitive function, and reducing inflammation (Jagim et al. 2023). Several probiotic strains have been identified for their potential to enhance mental health and alleviate psychological stress. For instance, *B. longum* 1714 has demonstrated stress‐reducing and memory‐enhancing effects in a study involving 22 healthy male participants (Allen et al. 2016). Similarly, *B. longum* BB536 has been studied for its ability to mitigate stress‐induced symptoms and improve overall mental well‐being (Xu et al. 2024; Orikasa et al. 2016). *B. bifidum* is recognized for its capacity to elevate mood and reduce stress levels, frequently included in probiotic supplements targeting mental health support (Li et al. 2023). *L. rhamnosus* has shown significant effects on anxiety and depression, with research indicating its ability to modulate the gut–brain axis, thereby influencing mood and cognitive function (Sharma et al. 2021). Studies have also indicated that *L. acidophilus* can help alleviate symptoms of anxiety and depression, often used in conjunction with other probiotics to enhance its mental health benefits (Liu, Walsh, and Sheehan 2019). *L. helveticus* has been associated with reducing symptoms of anxiety and depression, commonly combined with strains like *B. longum* to augment its mental health effects (Liu, Walsh, and Sheehan 2019). Lastly, *L. plantarum* has demonstrated positive effects on mood and cognitive function, frequently utilized in probiotic formulations designed to support mental health. These strains are part of a broader category known as psychobiotics, which specifically target mental health by influencing the gut–brain axis (Sharma et al. 2021). However, individual responses may vary, and more research is needed to determine specific strains, doses, and durations of supplementation for consistent effects on psychological stress in athletes. Probiotics should be considered as part of a holistic approach to mental well‐being, along with proper nutrition, adequate sleep, stress management strategies, and professional consultation when needed. It is crucial to recognize that probiotics are one aspect of supporting mental resilience and should not be considered a standalone solution (Mason et al. 2023).

### 5.6 Antioxidant Activity

**Table:** 

Key point 6: Overview of how probiotics may exhibit antioxidant activity
Production of antioxidant molecules Scavenging free radicals Enhancement of host antioxidant defenses Reduced oxidative stress Protection against lipid peroxidation Fermentation and short‐chain fatty acids (SCFAs)

Probiotics have been investigated for their potential antioxidant properties, aiming to combat oxidative stress resulting from strenuous exercise. Several mechanisms illustrate how probiotics may exhibit antioxidant activity, including the production of antioxidant molecules (enzymes and peptides), scavenging free radicals, enhancing host antioxidant defenses, reducing oxidative stress, protecting against lipid peroxidation, and contributing to antioxidant effects through the fermentation of dietary fibers (Wang et al. 2017). Certain probiotic strains can produce antioxidant substances, such as superoxide dismutase and peptides, which neutralize free radicals and reduce oxidative stress. Probiotics may directly scavenge free radicals, stimulate the host's own antioxidant defenses, and protect against lipid peroxidation, preserving cell membrane integrity. Additionally, probiotic fermentation of dietary fibers can produce short‐chain fatty acids (SCFAs), contributing to antioxidant effects in the gastrointestinal tract (Soheili, Alinaghipour, and Salami 2022). Clinical studies have explored the antioxidant effects of probiotics in conditions like inflammatory bowel diseases, metabolic disorders, and cardiovascular health, with promising results. However, further research is needed to understand specific mechanisms and optimal conditions for achieving antioxidant benefits. Studies have shown that probiotic supplementation can increase plasma antioxidant levels, neutralizing reactive oxygen species induced by intense physical activity (Pyne et al. 2015).

Several probiotic strains have been recognized for their antioxidant properties. Specifically, *Lactobacillus* and *Bifidobacterium* species have been extensively studied for their ability to scavenge free radicals and inhibit lipid peroxidation, with varying levels of efficacy (Feng and Wang 2020). Notably, certain Bifidobacteria strains (BF17‐4, BF52‐1, BF87‐3, BF88‐5) have demonstrated superior antioxidant activity and cell surface properties compared to *L. rhamnosus* GG (Cai et al. 2022). Despite not excelling in cell surface properties, *L. rhamnosus* GG is still acknowledged for its antioxidant capabilities (Cai et al. 2022). Bacillus strains are also noted for their antioxidant potential, attributed to the production of multiple biologically active molecules (Safronova, Skorochod, and Ilyash 2021). Additionally, *Propionibacterium freudenreichii* has exhibited significant antioxidant activity, achieving a maximum DPPH scavenging potential of 97.75% (Baku 2012). It is crucial to recognize that the antioxidant activity of probiotics can vary among strains, and while probiotics may contribute to antioxidant defenses, maintaining a well‐balanced diet rich in fruits, vegetables, and other antioxidant‐rich foods remains essential for overall health (Singh et al. 2023).

### 5.7 Nutrient Absorption

**Table:** 

Key point 7: Probiotics contribute to increased nutrient absorption through various mechanisms
Gut microbiota balance Improved gut barrier function Enhanced digestive enzyme activity Short‐chain fatty acid production Optimized nutrient metabolism

Emerging research indicates that probiotics, traditionally associated with digestive health, may have a role in enhancing nutrient absorption, particularly in athletes (Wang and Ji 2019). Probiotics contribute to increased nutrient absorption through various mechanisms. Firstly, they help maintain a balanced gut microbiome, which is crucial for optimal digestion and nutrient absorption. Disruptions in this balance can negatively affect nutrient absorption (Zhao et al. 2023b). Additionally, probiotics contribute to a strong gut barrier, preventing the leakage of undigested food particles and harmful substances into the bloodstream, which can interfere with nutrient absorption (Li et al. 2021b). Moreover, probiotics may stimulate the production of digestive enzymes, essential for breaking down nutrients into absorbable forms, thereby improving overall digestion and nutrient absorption (Assan et al. 2022). Furthermore, certain probiotic strains produce short‐chain fatty acids (SCFAs) during fermentation, which play a role in maintaining gut health and have been associated with increased absorption of certain nutrients, such as minerals (Al‐Qadami et al. 2022). Lastly, probiotics may influence the metabolism of nutrients, enhancing the bioavailability of vitamins and minerals, and ensuring a higher percentage of ingested nutrients are absorbed.

Research suggests that altering the microbiota profile through probiotics can impact nutrient absorption, especially in the small intestine. Probiotics can be a method to relieve malnutrition by influencing the gut microbiome (Ruigrok, Weersma, and Vich Vila 2023). Various probiotic strains have been demonstrated to augment nutrient absorption (Varvara and Vodnar 2024). *L. acidophilus* is recognized for its ability to enhance the assimilation of several nutrients, including B vitamins and minerals such as iron and calcium (Ballini et al. 2019). *L. gasseri* exerts an influence on energy balance and fat storage through the regulation of nutrient absorption (Nadeem et al. 2024). *L. plantarum* facilitates the breakdown of complex macronutrients and contributes digestive enzymes, thereby potentially improving nutrient absorption (Wang and Ji 2019; Kwoji et al. 2021). *B. lactis* has been observed to increase the bioavailability of minerals like calcium and magnesium, which may lead to enhanced nutrient absorption (Varvara and Vodnar 2024). *B. breve* similarly plays a role in regulating nutrient absorption, impacting energy balance and fat storage (Nadeem et al. 2024). *S. boulardii* is noted for its capacity to enhance nutrient absorption by preserving the integrity of the intestinal barrier (Terciolo, Dapoigny, and Andre 2019). However, individual responses may vary, and the specific strains of probiotics, as well as overall gut health, can influence outcomes. Athletes, with increased nutrient needs due to higher physical activity levels, may benefit from maintaining a healthy gut microbiome through probiotic consumption for improved nutrient absorption (Pyne et al. 2015).

### 5.8 Gut–Brain Axis

**Table:** 

Key point 8: Actions through which probiotics can influence the gut–brain axis in athletes
Neurotransmitter production Immunomodulation Short‐chain fatty acid production Hormonal regulation Reduced inflammation

The gut–brain axis, a two‐way communication system connecting the central nervous system (CNS) and the enteric nervous system, involves neural, hormonal, and immune pathways (Makris et al. 2021). Probiotics, which influence the gut microbiota, may modulate this axis, impacting mood and cognitive function, which are crucial for athletes' mental well‐being and performance (Clark and Mach 2016). Probiotics can influence the gut–brain axis in athletes through neurotransmitter production, immunomodulation, short‐chain fatty acid production, hormonal regulation, and reducing inflammation. Probiotics promote a healthy gut microbiome, influencing the production and release of neurotransmitters (such as serotonin, dopamine, and GABA), potentially affecting an athlete's mental state and stress response (Clark and Mach 2016). Probiotics affect the gut immune system, regulating inflammation that can influence the brain and behavior, indirectly impacting the gut–brain axis (D'Mello et al. 2015). Probiotics produce short‐chain fatty acids (SCFAs) with retroactive effects, influencing the gut–brain axis by maintaining the gut barrier and having anti‐inflammatory properties (Al‐Qadami et al. 2022). Probiotics influence hormone production, potentially affecting stress hormones like cortisol, crucial for athletes in balancing the stress response (Wu et al. 2023). Probiotics may reduce systemic inflammation, potentially benefiting the gut–brain axis, especially during intense training or competition (Clark and Mach 2016). Recent research highlights the gut–brain axis as a partnership through the vagus nerve (Bonaz, Sinniger, and Pellissier 2019). Probiotics, influencing the gut–brain axis, may boost mood and cognitive function and reduce stress and anxiety. For instance, a study on Alzheimer's patients showed improved cognitive scores after consuming probiotic‐rich milk (Breit et al. 2018).

Various probiotic strains have been identified to exert modulatory effects on the gut–brain axis (Lee, Lee, and Hur 2022). Specifically, *L. rhamnosus* has been demonstrated to alleviate anxiety and depression‐like symptoms by influencing the GABAergic system within the brain (Kumar et al. 2024; Rajanala, Kumar, and Chamallamudi 2021). Additionally, *L. acidophilus* has been found to mitigate stress and anxiety by modulating the hypothalamic–pituitary–adrenal (HPA) axis, which is intricately linked to the gut–brain axis (Yang et al. 2020; Rahmannia et al. 2024). Studies have also shown that *B. longum* can enhance mood and diminish anxiety by modifying neurotransmitter levels in the brain and regulating the gut–brain axis (Kumar et al. 2024; Ansari et al. 2023). Research further indicates that *B. breve* can enhance cognitive function and reduce stress‐related behaviors by exerting influence on the gut–brain axis (Snigdha et al. 2022; Rahmannia et al. 2024). Lastly, *Limosilactobacillus reuteri* has been demonstrated to modulate the gut–brain axis and facilitate serotonin synthesis, which is essential for mood regulation and overall brain health (Nobile, Giardina, and Puoci 2022). The relationship between probiotics and the gut–brain axis is an active research area, with varied athlete responses. Further research is needed to determine specific probiotic strains, doses, and supplementation durations for consistent and significant effects (Akbari et al. 2016).

### 5.9 Bone Health

**Table:** 

Key point 9: Probiotics may indirectly impact bone health through various pathways
Nutrient absorption Vitamin production Reduced inflammation Short‐chain fatty acids (SCFAs) Hormonal regulation

The correlation between probiotic consumption and bone health in athletes is an emerging field of study (Schepper et al. 2017). While a definitive connection has not been fully established, evidence suggests that probiotics may indirectly influence bone health through various pathways such as nutrient absorption, vitamin production, reduced inflammation, short‐chain fatty acids (SCFAs), and hormonal regulation.

Probiotics contribute to maintaining a healthy gut microbiome, which enhances the absorption of minerals essential for bone health, such as calcium and magnesium (Varvara and Vodnar 2024). Specific probiotic strains are capable of synthesizing vitamin K2, which plays a crucial role in bone metabolism by regulating calcium and promoting bone mineralization (Akbari and Rasouli‐Ghahroudi 2018). Indirectly, probiotics may support bone health by modulating the immune response and reducing chronic inflammation, which can have adverse effects on bone density (D'Amelio and Sassi 2018). Additionally, probiotics produce SCFAs, including butyrate, which have beneficial effects on bone health by influencing bone metabolism and maintaining a balance between bone formation and resorption (Markowiak‐Kopec and Slizewska 2020). Furthermore, probiotics may influence hormonal pathways related to bone health, affecting the production of hormones such as parathyroid hormone (PTH) and calcitonin (Schepper et al. 2017).

The relationship between probiotic strains and bone health is substantiated by numerous studies that highlight the positive impact of specific probiotic strains on various aspects of bone health. Empirical evidence from both clinical trials and preclinical research underscores the beneficial effects of probiotics on skeletal integrity (McCabe and Parameswaran 2018; Collins et al. 2017). For instance, probiotics such as VSL#3 and *L. rhamnosus* GG have been identified as advantageous for bone health (Cooney et al. 2020). The efficacy of individual probiotic strains in enhancing bone metabolism has been demonstrated; *B. lactis* Probio‐M8, for example, has been shown to augment bone formation and diminish bone resorption (Zhao et al. 2023a). Conversely, a synergistic effect on bone health parameters has been observed with a combination of three *Lactobacillus* strains ( *L. paracasei* DSM 13434, *L. plantarum* DSM 15312, and *L. plantarum* DSM 15313) in a multicenter, randomized, double‐blind, placebo‐controlled trial, suggesting that multiple strains may confer additional benefits (Cronin et al. 2022). A recent study conducted by Rizzoli and Biver (2020) suggests that probiotics may safeguard bone health by preventing bone loss associated with estrogen deficiency, diabetes, or glucocorticoid treatments (Rizzoli and Biver 2020). Another study involving laboratory mice discovered that supplementation with *L. rhamnosus* GG stimulated the growth of gut bacteria producing butyrate, thereby promoting bone growth (Tyagi et al. 2018). However, it is essential to note that these findings are based on animal studies, and further research is necessary to validate them in human subjects.

Despite theoretical connections, establishing a clear and direct link between probiotic consumption and bone health in athletes requires additional investigation (Schepper et al. 2017). Their effects may vary depending on factors such as probiotic strains, individual gut microbiota composition, and other variables. Athletes concerned about bone health should prioritize a well‐balanced diet rich in essential nutrients, including calcium, vitamin D, vitamin K2, and magnesium (Samoilov et al. 2023).

### 5.10 Recovery and Muscle Soreness

**Table:** 

Key point 10: Potential mechanisms of impact of probiotic supplementation on recovery
Reduced inflammation Enhanced immune function Gut–brain axis interaction Improved nutrient absorption Regulation of gut permeability

Exploration of the potential advantages of probiotics for aiding recovery and alleviating muscle soreness in athletes is an evolving domain. Although definitive evidence is not yet available, several studies indicate potential benefits of probiotic supplementation on recovery (McDermott et al. 2022). The mechanisms that might contribute to this include reducing inflammation, enhancement of immune function, interaction with the gut–brain axis, improvement in nutrient absorption, and regulation of gut permeability.

Probiotics' ability to reduce inflammation may help alleviate exercise‐induced inflammation and oxidative stress, thereby decreasing the severity of muscle soreness (Zaib, Hayat, and Khan 2024). By maintaining a healthy balance of gut bacteria, probiotics could bolster immune function, potentially reducing the incidence of illnesses and facilitating faster recovery (Dahiya and Nigam 2022). Impacting the gut–brain axis, probiotics may influence mood and stress levels, indirectly contributing to diminished muscle soreness as mental well‐being plays a role in the recovery process (Cammisuli et al. 2022). Furthermore, probiotics can aid in nutrient absorption, supporting various recovery processes such as muscle repair and rebuilding (Marttinen et al. 2020). They might also sustain the integrity of the gut lining, thus reducing intestinal permeability and the risk of harmful substances leaking into the bloodstream, potentially leading to decreased systemic inflammation and muscle soreness (DiMattia et al. 2023).

The potential of probiotic strains in facilitating recovery and mitigating muscle soreness has been the subject of scientific investigation. Notably, *Bacillus coagulans* GBI‐30, 6086, has been found to exert anti‐inflammatory effects, a critical factor in muscle soreness and recovery. These probiotics modulate the immune system and decrease inflammatory cytokines such as IL‐6, thereby contributing to the alleviation of muscle pain and acceleration of post‐exercise recovery (Jäger et al. 2016b; Jäger et al. 2016a). *L. plantarum* has been implicated in anti‐inflammatory responses, potentially aiding in the reduction of muscle soreness post‐exercise (Fu et al. 2021). *L. casei* has been associated with enhanced immune function, which may assist in the recovery process among athletes (Zhang, Zhang, and Li 2023). *L. acidophilus* , recognized for its health‐promoting properties, may contribute to overall recovery and the reduction of muscle soreness (Zhang, Zhang, and Li 2023). *L. helveticus* has been explored for its potential to diminish muscle damage, potentially enhancing recovery from strenuous physical activity (Iwasa and Aoi 2017). Additionally, research indicates that *B. breve* may bolster immune function, thereby potentially aiding in recovery from exercise‐induced stress (Marttinen et al. 2020; Toda et al. 2020).

A position statement from the International Society of Sports Nutrition connects certain anti‐inflammatory probiotic strains with enhanced recovery from muscle‐damaging exercise (Jäger et al. 2019a). However, more research is necessary to establish a definitive cause‐and‐effect relationship between probiotics and improved recovery or reduced muscle soreness in athletes. Individual responses may vary, and the effectiveness of probiotics may hinge on factors such as specific strains, overall health status, and training intensity. Athletes should consider probiotic supplementation as part of a holistic recovery approach, taking into account factors like nutrition, hydration, sleep quality, and other recovery strategies (Pyne et al. 2015).

### 5.11 Skeletal Muscle Metabolism

**Table:** 

Key point 11: Probiotics play a role in modulating skeletal muscle metabolism through a number of potential mechanisms
Inflammation modulation Short‐chain fatty acids (SCFAs) Insulin sensitivity Muscle protein synthesis Hormonal regulation

The association between probiotics consumption and skeletal muscle metabolism is a burgeoning area of interest. Emerging evidence indicates that probiotics, by influencing the gut microbiota, may play a role in regulating skeletal muscle metabolism through potential mechanisms including inflammation modulation, short‐chain fatty acid (SCFA) production, insulin sensitivity, muscle protein synthesis, and hormonal regulation (Okubo et al. 2018).

Probiotics' anti‐inflammatory properties may indirectly bolster healthy skeletal muscle metabolism by alleviating chronic inflammation, which can contribute to muscle wasting and impaired function (Nucci et al. 2023). Through the production of SCFAs, probiotics can provide an energy source for muscle cells, thereby influencing metabolic health and potentially affecting skeletal muscle function (Cheng, Liu, and Ling 2022). Probiotics may also impact insulin sensitivity by modulating the gut microbiota, potentially enhancing glucose metabolism crucial for providing energy to skeletal muscles during exercise (Rad et al. 2017). Moreover, probiotics might influence the delicate balance between muscle protein synthesis and breakdown, which is essential for muscle growth and repair, particularly in athletes engaged in regular physical activity (Marttinen et al. 2020). Additionally, probiotics can affect the production and regulation of hormones related to metabolism, potentially influencing muscle growth and metabolism.

Certain probiotic strains have been demonstrated to augment the expression of genes pertinent to muscle health, including CCT, Pink1, Parkin, and Atg genes, which are critical for the maintenance and function of muscle cells (Hawrysh et al. 2023). Systematic reviews and meta‐analyses have substantiated the safety and efficacy of probiotics in enhancing muscle function, particularly in geriatric populations (Prokopidis et al. 2023; Zeng, Luo, and He 2024). *Lactiplantibacillus plantarum* has been found to improve exercise performance and mitigate fatigue, effects that can be attributed to its influence on muscle metabolism and function (Cheng et al. 2023). *L. plantarum* PS128 has been shown to ameliorate oxidative stress, inflammation, and performance in triathletes subjected to high‐intensity exercise, thereby highlighting its role in muscle metabolism (Huang et al. 2019). *L. fermentum* VRI‐003 has demonstrated the capacity to enhance mucosal immunity in endurance athletes, which may indirectly modulate muscle metabolism by diminishing inflammation and oxidative stress (Zhang, Zhang, and Li 2023; Aykut et al. 2024). A recent review by Lin et al. (2022) suggests that the gut microbiota can interact with skeletal muscle, regulating various processes that affect host physiology (Li, Jin, and Fan 2022). Although the exact mechanisms are not fully understood, it is believed that probiotics may alter the gut microbiota and associated metabolites, thereby supporting human skeletal muscle metabolism and function. Studies by Chen et al. (2022b) and Zhang, Zhang, Zhang, and Li (2023) propose that probiotics may enhance muscle glucose homeostasis, energy expenditure, protein synthesis, and physical activity by regulating intestinal permeability and metabolites. However, since these studies were conducted on animals, further research is necessary to validate these findings in humans (Zhang, Zhang, and Li 2023; Chen et al. 2022b).

It is essential to recognize that while these potential mechanisms suggest a connection between probiotics and skeletal muscle metabolism, more research is required to establish a clear cause‐and‐effect relationship. The effects of probiotics may vary depending on specific strains, individual gut microbiota composition, and other influencing factors (Cammisuli et al. 2022).

### 5.12 Fat Metabolism

**Table:** 

Key point 12: Mechanisms of potential connections between probiotic consumption and fat metabolism in athletes
Microbiota composition Short‐chain fatty acids (SCFAs) Inflammation reduction Insulin sensitivity Hormonal regulation

While the direct correlation between probiotic consumption and fat metabolism in athletes remains incompletely understood, emerging evidence suggests potential associations through various mechanisms (Cai et al. 2023), including microbiota composition, short‐chain fatty acid (SCFA) production, inflammation reduction, insulin sensitivity, hormonal regulation, and energy expenditure.

Probiotics may influence the overall composition of the gut microbiota by fostering a healthy balance of gut bacteria, which could potentially impact fat metabolism (Kamal et al. 2023). The production of SCFAs by probiotics, particularly through the fermentation of dietary fibers, is linked to metabolic benefits, such as modulating fat metabolism by influencing lipid storage and utilization (Bongiovanni, Yin, and Heaney 2021). Probiotics' anti‐inflammatory properties may indirectly support healthy fat metabolism by addressing chronic inflammation, often associated with metabolic dysfunction (Plaza Díaz et al. 2014). Additionally, probiotics might affect insulin sensitivity, a crucial factor in regulating glucose and lipid metabolism, potentially enhancing the body's ability to utilize fats for energy during endurance activities (Sáez‐Lara et al. 2016). Furthermore, probiotics' influence on the gut microbiota can interact with the endocrine system, affecting the production and regulation of hormones like leptin and ghrelin, which play roles in appetite regulation and fat metabolism (Jia et al. 2023). Some research suggests that probiotics could impact energy expenditure, potentially affecting fat oxidation and proving beneficial during prolonged endurance exercise (Ormsbee, Bach, and Baur 2014).

Recent studies by Wiciński et al. (2020) and Yadav et al. (2013) propose that probiotics may decrease body weight and BMI by inhibiting dietary fat absorption, increasing fat excretion, and modulating the release of appetite‐regulating hormones (Yadav et al. 2013; Wiciński et al. 2020). Certain bacteria, particularly those from the Lactobacillus family, have been identified to function in this manner. Yoo and Kim (2016) suggest that probiotics might combat obesity by modulating gut bacteria and associated metabolites, thereby supporting human skeletal muscle metabolism and function (Yoo and Kim 2016). Various probiotic strains, including *L. acidophilus* , *B. animalis* subspecies *lactis*, and *L. reuteri* , have been the subject of research examining their influence on fat metabolism. Studies have indicated that certain strains may contribute to a reduction in total cholesterol (TC) and low‐density lipoprotein cholesterol (LDL‐C), particularly among individuals with elevated baseline cholesterol levels (Gadelha and Bezerra 2019; Cho and Kim 2015; Million et al. 2012). Additionally, other strains such as *B. breve* , *B. longum* , *S. thermophilus* , *L. delbrueckii* , and *L. casei* have been shown to exert beneficial effects on fat metabolism (Maftei et al. 2024). The impact of probiotic strains on lipid metabolism exhibits strain‐specific variations. For instance, *L. rhamnosus* and *L. johnsonii* have demonstrated efficacy in diminishing abdominal adiposity and body mass. Conversely, *B. animalis* has been associated with enhanced hepatic function and lipid metabolism (Chen et al. 2022a; Wu et al. 2022b).

Research conducted on animal models has demonstrated that probiotics can effectively prevent and mitigate obesity and associated chronic conditions by modulating lipid, glucose, and cholesterol metabolism (Tang et al. 2021; Wang et al. 2024b). Clinical investigations and meta‐analyses, however, have yielded inconsistent findings, with some suggesting that probiotics can substantially decrease total cholesterol (TC) and low‐density lipoprotein cholesterol (LDL‐C) levels, particularly in subjects with elevated baseline cholesterol concentrations (Cho and Kim 2015). However, further research is necessary to validate these findings, as the effects of probiotics on fat metabolism may vary depending on the strain, and individual responses could differ based on factors such as baseline gut microbiota composition, diet, and overall health. Athletes interested in the potential benefits of probiotics for fat metabolism should consider integrating them into a comprehensive strategy that includes proper nutrition, training, and recovery practices (Marttinen et al. 2020).

### 5.13 Body Weight

**Table:** 

Key point 13: Potential mechanisms of the correlation between probiotic consumption and body weight adjustment in athletes
Regulation of appetite Modulation of metabolism Influence on fat mass Anti‐inflammatory effects Short‐chain fatty acid production Improved nutrient absorption

The correlation between probiotic consumption and body weight adjustment in athletes is an evolving area of study, with potential mechanisms suggested by emerging evidence (Mekkes et al. 2014), including regulation of appetite, modulation of metabolism, influence on fat mass, anti‐inflammatory effects, short‐chain fatty acid production, and improved nutrient absorption.

Probiotics may impact the gut–brain axis, potentially affecting appetite and satiety signals, and regulating hormones such as ghrelin and leptin (Lean and Malkova 2016). They might also play a role in metabolic regulation, influencing energy utilization and storage, which could include modulation of insulin sensitivity (Blandino et al. 2016). Some research indicates that probiotics could contribute to reducing fat mass, possibly through alterations in gut microbiota composition that affect energy extraction and fat storage (Sanchez, Panahi, and Tremblay 2015). By reducing inflammation, probiotics may contribute to improved metabolic health, potentially supporting weight management efforts (Torres et al. 2019). Additionally, the production of short‐chain fatty acids by probiotics has been associated with metabolic benefits, including appetite regulation and energy metabolism (Byrne et al. 2015). Furthermore, probiotics can enhance nutrient absorption, impacting overall nutritional status and energy balance, which may influence body weight.

The ingestion of probiotics has been linked to a notable decrease in body weight and Body Mass Index (BMI). For example, one study revealed that the consumption of probiotics resulted in a reduction of body weight by 0.59 kg and BMI by 0.49 kg/m^2^ (Zhang, Wu, and Fei 2015). Another investigation demonstrated that a 12‐week supplementation with a multi‐strain probiotic (UB0316) significantly lowered BMI (Sudha et al. 2019). Probiotic strains have been shown to exert multiple effects on body weight and weight management. Probiotics, particularly strains such as *L. rhamnosus* , can modulate the gut microbiota, which in turn can influence body weight, glucose, and fat metabolism (Mazloom, Siddiqi, and Covasa 2019). This modulation aids in enhancing metabolic health and diminishing obesity‐related complications (Cao et al. 2024). Certain strains within the genera *Lactobacillus* and *Bifidobacterium* have exhibited the most promising results in reducing body weight. This underscores the significance of selecting the appropriate strains for specific health outcomes (Álvarez‐Arraño and Martín‐Peláez 2021).

The relationship between probiotics and body weight is complex, and study results are varied. While certain studies suggest a potential role for probiotics in weight management, others show no significant impact. Additionally, the effects of probiotics can be strain‐specific, and individual responses may vary depending on factors such as diet, exercise habits, and overall health (Magne et al. 2020). Athletes considering probiotics for weight management should integrate them into a comprehensive strategy that includes proper nutrition, regular physical activity, and overall wellness practices. Consulting with healthcare professionals or sports nutrition experts is advisable to align probiotic supplementation with specific needs and goals. It is crucial to view probiotics as one component of a holistic approach to health and performance, rather than relying solely on them for weight management (Coqueiro et al. 2017).

## 6 The Main Benefits of Probiotic Consumption in Amateur Athletes

**Table:** 

Key point 14: The main benefits of probiotic consumption in amateur athletes
Gut health Immune system support Recovery Energy metabolism Gut–brain axis interaction Digestive comfort

Probiotic consumption holds promise for amateur athletes, though ongoing research is refining our understanding of its benefits. These benefits encompass fostering a healthy balance of the gut microbiota, which positively impacts digestion, nutrient absorption, and overall gut health. Probiotics may bolster immune function, crucial for staving off infections, especially during rigorous training (Coqueiro et al. 2017). They might also aid in reducing inflammation and facilitating recovery, potentially shortening the time needed between workouts for amateurs engaged in regular training (Marttinen et al. 2020). Some studies suggest that probiotics could influence energy metabolism and nutrient utilization, pertinent for athletes aiming for optimal energy levels during training and competitions (Wang and Ji 2019). Additionally, probiotics may interact with the gut–brain axis, contributing to improved mental resilience and focus in amateur athletes, affecting mood, stress response, and overall mental well‐being (Clark and Mach 2016). Finally, probiotics may alleviate gastrointestinal issues such as bloating, gas, and irregular bowel movements, frequently encountered by athletes engaging in regular exercise (Pyne et al. 2015).

The literature indicates that probiotics have demonstrated efficacy in enhancing digestion, boosting immune function, reducing gastrointestinal symptoms, and positively influencing performance‐related factors in amateur athletes (Nichols 2007). Different probiotic strains may exert distinct effects, highlighting the importance of consuming a variety of strains for comprehensive benefits (Díaz‐Jiménez et al. 2021). Various probiotic strains are commonly consumed by amateur athletes. *L. plantarum* has been demonstrated to enhance both anaerobic and aerobic endurance, diminish fatigue, and mitigate inflammation and oxidative stress in amateur athletes (Paiva et al. 2022). *L. acidophilus* is recognized for its advantages in optimizing gut health and immune function, which are essential during training (Zhang, Zhang, and Li 2023). *L. casei* also confers benefits to gut health and immune function, and it has been investigated for its potential to lower the frequency of upper respiratory tract infections in amateur athletes (Gleeson et al. 2011). *B. bifidum* is efficacious in modulating the immune system and enhancing gut health, which can assist amateur athletes in sustaining their performance during rigorous training (Łagowska and Bajerska 2021). *S. thermophilus* is frequently utilized in conjunction with other probiotics and is noted for its contribution to improving gut health and immune function during training (Aykut et al. 2024; Leite et al. 2019; Shing et al. 2014). Integrating naturally fermented foods into the diet can also foster diverse and healthy gut microbiota. Responses to probiotics among amateur athletes can vary, necessitating some trial and error to find the right strains and doses. When combined with a well‐balanced diet, proper hydration, and suitable training strategies, probiotic consumption can contribute to overall well‐being and athletic success for amateur athletes (Di Dio et al. 2023).

## 7 The Main Benefits of Probiotic Consumption in Professional Athletes

**Table:** 

Key point 15: The main benefits of probiotic consumption in professional athletes
Gut health and function Immune support Recovery and reduced inflammation Mental well‐being and focus Nutrient utilization and energy metabolism Digestive comfort

Probiotic consumption is garnering attention among professional athletes due to its potential benefits across various domains (Sivamaruthi, Kesika, and Chaiyasut 2019). Probiotics play a crucial role in maintaining a healthy gut microbiome, which aids in digestion, nutrient absorption, and the alleviation of gastrointestinal issues often experienced by athletes undergoing rigorous training (Huang et al. 2020a). They may also fortify the immune system in professional athletes, reducing the susceptibility to illnesses brought on by the physical strain of intense training (Pyne et al. 2015). Moreover, probiotics contribute to mitigating exercise‐induced inflammation, thereby promoting faster recovery between training sessions—a vital consideration for professionals with demanding schedules (Marttinen et al. 2020). Additionally, they may influence the gut–brain axis, impacting mood, the stress response, and focus—critical factors for professional athletes (Thangaleela et al. 2022). Furthermore, probiotics have the potential to enhance nutrient utilization and energy metabolism, potentially optimizing the conversion of nutrients into energy during exercise. Probiotics can also alleviate digestive discomfort resulting from factors such as high‐calorie diets, travel, and stress in professional athletes (Kumar et al. 2023).

The International Society of Sports Nutrition recognizes the potential probiotic benefits for professional athletes, including improved body composition, lean body mass, normalized testosterone levels, reduced cortisol levels, decreased exercise‐induced lactate buildup, and enhanced neurotransmitter synthesis, cognition, and mood (Jäger et al. 2019a).

Professional athletes frequently incorporate particular probiotic strains into their regimens to augment performance, expedite recovery, and bolster overall well‐being (Díaz‐Jiménez et al. 2021; Jäger et al. 2019b). *L. casei* Shirota (LcS) is recognized for its efficacy in mitigating respiratory symptoms, thereby aiding athletes in sustaining peak respiratory health amid rigorous training and competitive events (Gleeson et al. 2011). *L. plantarum* is a component of a five‐strain probiotic formulation that also encompasses HN019, NCFM, La‐14, BL‐04, and LPC‐37, all of which have been clinically validated to promote gastrointestinal health and, consequently, athletic performance (Huang et al. 2019; Harris et al. 2022). *L. acidophilus* is employed to bolster respiratory health and overall immune function, which is critical for elite athletes to maintain health and peak performance (Sivamaruthi, Kesika, and Chaiyasut 2019). The innovative strain *B. animalis* subsp. *lactis* BB‐12 has been patented for its potential to enhance athletic performance and recovery (Dong et al. 2020). *B. animalis* subsp. *lactis* is utilized to diminish the risk of respiratory infections, a significant concern for elite athletes who are at an increased risk due to the intensity of their training (Di Dio et al. 2022). *B. longum* OLP‐01, isolated from the gut of an Olympic weightlifting champion, has been engineered to augment athletic performance and recovery (Lin et al. 2022; Huang et al. 2020a). The probiotic strain PS128 has demonstrated the ability to reduce exercise‐induced damage and fatigue in athletes engaged in high‐intensity activities, while also facilitating muscle recovery and enhancing endurance performance (Huang et al. 2019; Huang et al. 2020b). *Veillonella atypica* , identified in the gut microbiomes of elite athletes, is noted for its capacity to metabolize lactic acid generated during strenuous exercise, transforming it into propionate, which may enhance exercise tolerance (Scheiman et al. 2019; Gross et al. 2024).

Given the variability in individual responses to probiotics, experimentation with different strains and doses is necessary. It is advisable for professional athletes to consult healthcare professionals or sports nutritionists to tailor probiotic supplementation to their specific needs and goals. Probiotic consumption can form part of a comprehensive strategy that includes proper nutrition, hydration, training, and recovery, taking into account individual factors (Coqueiro et al. 2017).

## 8 Consumption of Probiotics During Training

**Table:** 

Key point 16: Consumption of probiotics during training
Daily supplementation Pre‐workout consumption Post‐workout recovery During endurance exercise Balanced diet complement Travel considerations

Athletes are increasingly integrating probiotics into their training regimens, recognizing the potential health and performance benefits they offer and adopting diverse approaches. Some athletes incorporate daily probiotic consumption as part of their routine to uphold a balanced gut microbiome and overall health throughout training (Marttinen et al. 2020). Pre‐workout probiotic intake aims to address factors like digestive comfort and nutrient absorption during exercise, although determining the optimal timing requires further investigation. Including probiotics in post‐workout nutrition may aid recovery by bolstering the immune system and mitigating inflammation following exercise‐induced stress (Jäger et al. 2016b). Athletes engaging in prolonged endurance activities often turn to probiotics to support gut function and minimize the risk of gastrointestinal distress. Probiotic‐rich foods, such as yogurt, can complement the diets of athletes, providing additional nutrients alongside probiotics (Jäger et al. 2016b). When facing changes in diet, sleep, and routine during travel, athletes may consider incorporating probiotics to maintain gut health.

Recent research suggests that specific probiotic strains can improve nutrient absorption and enhance immune function, thereby reducing the severity and duration of upper respiratory tract infections in athletes during training (Bielik et al. 2022). According to the reference materials, various probiotic strains commonly present in different supplements and fermented foods can offer advantages to athletes during training. The *Lactobacillus* genus encompasses several strains that are advantageous for gastrointestinal health and have been investigated for their potential to alleviate gastrointestinal issues and enhance immune function during periods of high‐stress training (Pyne et al. 2015). Analogous to *Lactobacillus*, *Bifidobacterium* strains are well‐documented and recognized for their support of digestive health and immune function during training regimens (Pyne et al. 2015). The *S. boulardii* strain is distinguished for its capacity to bolster gut health and has demonstrated efficacy in reducing the risk of antibiotic‐associated diarrhea and other gastrointestinal disturbances (Gopalan et al. 2023; Pais et al. 2020). *B. subtilis* is notable for its ability to withstand the harsh environment of the stomach and reach the intestines, where it can contribute to digestive health and immune function in athletes (Townsend et al. 2018). *S. thermophilus* , frequently found in yogurt and other fermented dairy products, is recognized for its role in supporting gut health and immune function during training sessions (Díaz‐Jiménez et al. 2021). However, optimal timing and individual responses to probiotics vary, necessitating experimentation with different strains and doses. While generally safe, athletes should take into account personal factors and preferences, recognizing that further research is needed to establish specific guidelines for probiotic consumption during training across different athletic contexts (Bielik et al. 2022).

## 9 Consumption of Probiotics During Competition

**Table:** 

Key point 17: Consumption of probiotics during competition
Immune support Gastrointestinal health Stress and anxiety management Preventing travel‐related issues Post‐competition recovery

The consumption of probiotics during sports competitions presents potential benefits for athletes, although individual responses may vary. Key advantages include maintaining gut microbiota balance, which can reduce the risk of infections amidst competition‐induced stress and related factors (Colbey et al. 2018). Furthermore, probiotics may promote digestive health, potentially mitigating exercise‐induced gastrointestinal issues during competitions (Wilson 2022). Additionally, they may contribute to managing mood and the stress response, crucial elements during intense competition. Probiotics can also aid in maintaining gut health and preventing digestive issues for athletes traveling during competitions (Bodke and Jogdand 2022). Moreover, consuming probiotics after intense competition may facilitate recovery by supporting immune function, reducing inflammation, and promoting gut health.

Research suggests potential benefits such as reduced cortisol levels, improved body composition, heightened response to stressors, and enhanced neurotransmitter synthesis. However, further research is necessary to determine the specific role of probiotics in athletes' nutrition during competitions. During competitive events, athletes may benefit from incorporating various probiotic strains into their regimen to enhance performance and overall well‐being. *L. plantarum* PS128 has been demonstrated to enhance both anaerobic and aerobic endurance, diminish fatigue, and mitigate inflammation and oxidative stress in athletes during competition (Marttinen et al. 2020; Huang et al. 2019). *L. acidophilus* confers benefits to digestive health and immune function, which are paramount during periods of high‐stress competition (Díaz‐Jiménez et al. 2021). *B. lactis* , recognized for its immune‐enhancing properties and capacity to improve gut health, can assist athletes in sustaining performance by reducing the risk of gastrointestinal and respiratory complications (Wosinska et al. 2019). *Akkermansia muciniphila* is particularly distinguished for its role in immune regulation and has been identified for its potential advantages in athletes during competition (Marttinen et al. 2020). *S. boulardii* is effective in preventing and treating diarrhea, a common issue during intense competitions (Wosinska et al. 2019; Czerucka and Rampal 2002).

Given the variability in individual responses, athletes should experiment with different strains and dosages well in advance of competition, taking into account their preferences and seeking guidance from healthcare professionals for personalized advice. The optimal timing of probiotic consumption during competitions remains uncertain and requires individual experimentation (Di Dio et al. 2023). Athletes should opt for reputable probiotic sources, considering convenience and personal preferences, and consult with healthcare professionals for tailored advice based on the type, intensity, and overall health status of the competition. The integration of probiotics into competition strategies should be approached in a personalized and well‐informed manner (Pyne et al. 2015).

## 10 Probiotic Strains Consumed by Athletes

Athletes commonly consume specific probiotic strains for potential health and performance benefits. These include the following:

*Lactobacillus acidophilus* : Supports digestive health, enhances nutrient absorption, and boosts immune function. Found in probiotic supplements and fermented foods like yogurt (Hussien, Abd‐Rabou, and Saad 2022).

*Lactobacillus rhamnosus* : Associated with improved exercise recovery, reduced muscle damage, and lower incidence of respiratory infections. Present in yogurt, kefir, and fermented foods (Mathipa‐Mdakane and Thantsha 2022).

*Lactobacillus rhamnosus* GG (LGG): LGG has been found to significantly ameliorate gastrointestinal discomfort during training and augment immune function (Sivamaruthi, Kesika, and Chaiyasut 2019; Yan and Polk 2012).

*Lactobacillus casei* : Believed to enhance immune function and reduce upper respiratory tract infections. Found in yogurt and dairy products (Hill et al. 2018).

*Lactobacillus paracasei* : This strain is recognized for its advantages in optimizing gut health and immune function, which are essential for athletes engaged in intense training regimens (Lee et al. 2022).

*Lactobacillus plantarum* : Promotes a healthy gut balance, supports digestive health, and may reduce inflammation, aiding in muscle recovery. Common in various fermented foods (Huang et al. 2020b).

*Lactobacillus plantarum* PS128: This probiotic strain has been demonstrated to enhance anaerobic and aerobic endurance, diminish fatigue, and mitigate inflammation and oxidative stress in athletes, particularly among triathletes and half‐marathon runners (Huang et al. 2019).

*Lactobacillus fermentum* VRI‐003: This strain has been investigated for its effects on mucosal immunity in endurance athletes (Aykut et al. 2024).

*Bifidobacterium animalis* subsp. *animalis*: Improves gut health and reduces gastrointestinal issues. Present in the large intestines of mammals, including humans (Egan et al. 2018).

*Bifidobacterium animalis* subsp. *lactis*: Known for promoting a healthy gut flora, improving digestion, supporting immune function, reducing inflammation, and enhancing nutrient absorption. Found in fermented dairy products (Lin et al. 2024).

*Bifidobacterium lactis* Bi‐07 and *Bifidobacterium animalis* subsp. *lactis* Bl‐04: These strains have been shown to reduce the risk of infections and positively modulate immune responses in athletes (Maneerat et al. 2013; Morovic et al. 2017).

*Bifidobacterium longum* OLP‐01: This strain, isolated from the gut of an Olympic weightlifting champion, is specifically formulated to support athletic performance and recovery (Lin et al. 2022; Huang et al. 2020a).

*Akkermansia muciniphila* : This strain is noted for its role in immune regulation and is considered beneficial for athletes (Cani and de Vos 2017).

The International Society of Sports Nutrition recommends specific probiotic strains with proven benefits for athletes, including *L. rhamnosus* GG, *B. bifidum* W23, *B. lactis* W51, *E. faecium* W54, *L. acidophilus* W22, *L. brevis* W63, *L. lactis* W58, and *L. casei* Shirota (Sivamaruthi, Kesika, and Chaiyasut 2019). However, the effectiveness of these strains may vary based on the athlete's level of professionalism, training or competition period, and individual responses. More research is needed to determine optimal probiotic types, dosages, and supplementation durations for athletes. Athletes are advised to consult with a nutritionist before incorporating new probiotic strains into their exercise routine (Łagowska et al. 2022).

## 11 Commercial Brands of Probiotic Supplements for Athletes

Probiotic brands for amateur athletes include the following:

Garden of Life: offers probiotic supplements formulated for athletes, containing strains like *Lactobacillus acidophilus* , *Bifidobacterium animalis* subsp. *lactis*, and *Lactobacillus casei* (Dolan et al. 2017).

Culturelle: well‐known for various formulations, including those designed for digestive health and immune support in athletes, with strains like *Lactobacillus rhamnosus* and *Lactobacillus casei* (Abe et al. 2013).

Renew Life: provides probiotic formulas targeted toward athletes, supporting digestion and overall wellness with strains like *Lactobacillus acidophilus* and *Bifidobacterium animalis* subsp. *Lactis* (Quin et al. 2018).

Align: popular for Bifidobacterium‐infused supplements, promoting digestive health and a balanced gut microbiome in athletes (Ghelardi et al. 2023).

Probiotic brands for professional athletes include the following:

Thorne FloraSport 20B: specifically formulated for professional athletes, containing 20 billion Colony Forming Units (CFUs) per capsule and free from gluten, soy, and dairy.

Klaire Labs Ther‐Biotic Pro IBS Relief: suitable for athletes with digestive issues, providing 25 billion CFUs per capsule (Florencio et al. 2024).

Garden of Life Dr. Formulated Probiotics Fitbiotic: designed to support healthy digestion and metabolism, offering 50 billion CFUs per serving and free from gluten, soy, and dairy (Czajeczny, Kabzińska, and Wójciak 2021).

Klean Probiotic: a probiotic supplement formulated to support digestive health and bolster the immune system in athletes. Each capsule of Klean Probiotic contains 15 billion Colony Forming Units (CFUs) of probiotic strains from the *Lactobacillus* and *Bifidobacterium* genera, combined with a prebiotic base. The supplement encompasses 8 distinct probiotic strains, including *Lactobacillus rhamnosus* , *Lactobacillus lactis* , *Bifidobacterium bifidum* , and *Bifidobacterium longum* (Athlete 2024).

Onnit Total Gut Health: a comprehensive supplement designed to support digestive health and foster a healthy gut microbiome in athletes. The supplement includes multiple probiotic strains, such as *Saccharomyces Boulardii*, which are beneficial bacteria that contribute to maintaining a healthy gut microbiome. It also contains prebiotics, which are non‐digestible fibers that serve as a food source for probiotics, aiding in their proliferation. Additionally, digestive enzymes in this product facilitate the breakdown of macronutrients, enhancing the body's ability to absorb nutrients from food. The ingredient Betaine HCl helps increase stomach acid, which is crucial for proper digestion (Thomas 2024).

Sound Probiotics: a brand specializing in probiotic supplements tailored specifically for endurance athletes. The probiotic strains included in Sound Probiotics products are specifically selected for endurance athletes and include *Bifidobacterium bifidum* and *Bifidobacterium longum* strains. These strains are noted for their high‐level folate production and have been studied for their benefits in athletes. The products are formulated to address the needs of endurance athletes, who often require additional support for their digestive health, immune function, and recovery due to intense training regimens (The Feed 2016). While there is no probiotic supplement exclusively named for sports, athletes are encouraged to consider factors such as the variety and number of strains, colony‐forming units (CFUs), and specific health benefits when selecting a probiotic supplement that aligns with their overall gut health needs (Pyne et al. 2015).

## 12 Period of Use and Effect of Probiotic Supplements in Athletes

The duration of probiotic supplementation in athletes varies across different studies, ranging from short term (3 weeks) to long term (several months). Research has explored the effects of probiotic supplementation over periods as brief as 3 weeks. For example, Huang et al. demonstrated that supplementation with *L. plantarum* PS128 for 3 weeks enhanced anaerobic and aerobic endurance performance in athletes (Huang et al. 2019). Some studies have employed a 12‐week supplementation period. For instance, Towsend et al. investigated the effects of 12 weeks of daily single‐strain probiotic supplementation on the immune and hormonal profiles of male athletes (Townsend et al. 2018). A 2021 study highlighted the effectiveness of multi‐strain probiotics over single‐strain probiotics in achieving athletic performance goals, recommending a common long‐term supplementation period of 12 weeks (Schreiber et al. 2021). Another study in 2020 demonstrated the efficacy of multi‐strain Lactobacillus and Bifidobacterium probiotic cocktails over 11 weeks, reducing gastrointestinal symptoms and preserving gut barrier function during exercise. Additionally, there are studies that have examined the effects of probiotic supplementation over longer durations, such as several months. These studies typically focus on the cumulative benefits of probiotics on immune function and gut health (Marttinen et al. 2020). A 2016 article showed that daily probiotic supplementation over 3 months of exhaustive aerobic exercise reduced upper‐respiratory tract illnesses in trained athletes (Strasser et al. 2016).

The duration of probiotic use for athletes varies based on individual needs, health goals, and circumstances. Generally considered safe for long‐term use, probiotics are often incorporated into daily routines for ongoing health benefits. Incorporating probiotics into daily routines for weeks to months is suggested for observing positive effects on gut health. Following antibiotic use, a long course of probiotics is recommended to restore a healthy gut microbiome (Zhong et al. 2021). Probiotics may be beneficial during intense training or competition periods, contributing to a healthy gut balance. Some athletes may choose short‐term probiotic use leading up to specific events, but caution is advised when introducing new supplements close to competitions. Individual responses to probiotics vary, with noticeable benefits appearing quickly in some and requiring an extended period for others. Athletes are advised to consult with healthcare professionals to determine the most suitable duration of probiotic supplementation based on individual needs and health status (Pyne et al. 2015).

## 13 Boosters for Probiotic Supplements in Athletes

**Table:** 

Key point 18: Boosters for probiotic supplements in athletes
Prebiotics Fiber Polyphenols Fermented foods Resistant starch Synbiotics Vitamin D Minerals Lifestyle factors

While there is no specific supplement designed to directly “boost” probiotics, several factors and substances can support a healthy gut environment, thus enhancing the efficacy of probiotics in athletes (Liu et al. 2022). These include non‐digestible fibers found in foods like garlic, onions, leeks, bananas, asparagus, and chicory root, which promote the growth of beneficial gut bacteria (Kaur et al. 2021). Additionally, a diet rich in fiber from fruits, vegetables, whole grains, and legumes serves as a fuel source for beneficial gut bacteria. Moreover, antioxidant‐rich compounds in certain fruits, vegetables, teas, and red wine may positively impact gut bacteria (Aziz et al. 2024), while naturally fermented foods like yogurt, kefir, sauerkraut, kimchi, and miso introduce live beneficial bacteria into the gut. Furthermore, resistant starch, found in green bananas, legumes, and cooked and cooled potatoes, reaches the colon and serves as a substrate for beneficial bacteria and synbiotics, which are combinations of probiotics and prebiotics aiming for a synergistic effect to enhance the survival and activity of probiotics in the gut (Jiang et al. 2022). Some research indicates that vitamin D may influence the gut microbiome. Athletes, particularly those with restricted sun exposure, may derive benefits from vitamin D supplementation (Yoon, Kwon, and Kim 2021; Todd et al. 2015). Additionally, zinc is crucial for immune function and may also contribute to maintaining a healthy gut microbiome. Other minerals, such as magnesium and selenium, are also vital for overall health and may indirectly support probiotic balance (Varvara and Vodnar 2024; Barone et al. 2022). Meanwhile, lifestyle factors such as adequate sleep, stress management, and regular physical activity contribute to overall gut health. Chronic stress and poor sleep can negatively impact the gut microbiome, while exercise has been associated with a more diverse microbial community (Donati Zeppa et al. 2019).

It is crucial to recognize that the effectiveness of these factors and supplements can vary among athletes. Consulting with a healthcare professional, particularly for athletes with underlying health conditions, is advisable before starting any new supplement regimen containing probiotics (Maughan et al. 2018).

## 14 Conclusions

Probiotic supplementation shows promise for enhancing overall athlete performance by immune system regulation, skeletal muscle regeneration, inflammatory response control, energy metabolism, intestinal barrier function, and stress reduction. The strategy offers potential benefits in maintaining gut health, immune function, and aiding post‐exercise recovery while potentially enhancing mental well‐being. However, a personalized approach that takes individual differences into consideration is strong. Future research should prioritize investigating probiotics' long‐term effects, mechanisms of action, and efficacy in clinical populations, and establishing regulatory guidelines for their safe and effective use in sports nutrition. Despite the significant market value of probiotic supplements for athletes, further research is needed to ascertain their safety, identify beneficial strains, determine optimal dosages, standardize protocols, and explore their use as functional products in sports nutrition.

## Author Contributions

**Yousef Nami:** writing – review and editing (equal). **Anahita Barghi:** writing – review and editing (equal). **Mehdi Shahgolzari:** writing – original draft (equal). **Melika Salehian:** writing – original draft (equal). **Babak Haghshenas:** project administration (equal), writing – review and editing (equal).

## Consent

The authors have nothing to report.

## Conflicts of Interest

The authors declare no conflicts of interest.

## Data Availability

The data presented in this study are available on request from the corresponding author.
